# Successful desensitization in paclitaxel-induced anaphylaxis: The role of skin testing and environmental sensitization

**DOI:** 10.5415/apallergy.0000000000000198

**Published:** 2025-03-17

**Authors:** João Cardoso Lopes, Helena Pires Pereira, Catarina Mesquita Guedes, Carmelita Ribeiro, Ana Todo-Bom

**Affiliations:** 1 Allergy and Clinical Immunology Department, Coimbra Hospital and University Centre, Coimbra, Portugal; 2 Coimbra Clinical Academic Centre, Faculty of Medicine, University of Coimbra, Coimbra, Portugal

**Keywords:** Desensitization protocol, environmental sensitization, paclitaxel anaphylaxis, skin testing, taxane hypersensitivity

## Abstract

Hypersensitivity reactions (HSRs) to taxanes, such as paclitaxel, occur in 5% to 10% of patients and can involve immunoglobulin E (IgE) or non-IgE mechanisms. We report a 65-year-old male with lung cancer who developed anaphylactic shock during his second paclitaxel treatment. Positive intradermal tests raised the suspicion of an IgE-mediated reaction and, consequently, the possibility of prior sensitization through environmental exposure to *Taxus baccata* (European yew) pollen. The patient underwent a 17-step desensitization protocol with nab-paclitaxel, a formulation with a lower risk of HSRs, and successfully completed 6 additional treatment cycles without recurrence. This case highlights the role of skin testing, potential environmental factors in taxane allergies, and desensitization protocols as effective strategies to ensure safe and uninterrupted therapy.

## 1. Introduction

Hypersensitivity reactions (HSRs) are unintended, harmful responses to drugs at therapeutic doses, involving immunoglobulin E (IgE), non-IgE, or unclear mechanisms [1, 2]. HSRs inducing-drugs are usually avoided, but desensitization can be an option, when no effective alternatives exist, for both IgE- and non-IgE-mediated HSRs [3]. Taxanes, including paclitaxel and nab-paclitaxel, are widely used for cancer treatment, particularly gynecologic and lung malignancies. Paclitaxel, originally derived from the bark of the Pacific yew tree (*Taxus brevifolia*), became more sustainable following the discovery of precursors in the leaves of the European yew (*Taxus baccata*) [4]. Taxane HSRs can occur in 5% to 10% of patients, often during initial treatment cycles. These reactions may be non-IgE-mediated (from complement activation due to preservatives such as Cremophor EL or Polysorbate 80), or IgE-mediated responses to taxanes or excipients [5]. Recent studies have demonstrated IgE-mediated mechanisms in some taxane-induced HSRs, supported by skin tests and immunoassays. Furthermore, environmental exposure to *Taxus baccata* pollen has been hypothesized as a sensitizing factor for taxane allergies in certain populations living near yew trees [4, 6].

## 2. Case presentation

We present a case of a 65-year-old French male, with stage IVA lung squamous cell carcinoma, undergoing palliative chemotherapy with paclitaxel and carboplatin, as well as biological therapy with pembrolizumab. During his second chemotherapy cycle, paclitaxel was administered first, triggering a severe anaphylaxis within 5 minutes, marked by a systolic blood pressure drop to 60 mmHg, tachycardia (120 bpm), bronchospasm, oxygen saturation decline to 80%, loss of consciousness and sphincter incontinence. The infusion was stopped, and treatment included high-volume fluid resuscitation, systemic corticosteroids (200 mg hydrocortisone and 125 mg methylprednisolone), antihistamines, aminophylline, and two 0.5 mg intramuscular adrenaline doses. Oxygen at 15 L/min via a high-flow mask was delivered and stabilization occurred within 3 hours, with gradual consciousness recovery. No immediate reaction had occurred during the first infusion, but a delayed mucocutaneous response a week later suggested initial sensitization that progressed to severe hypersensitivity upon reexposure. The patient was subsequently referred for specialized drug allergy evaluation.

**Figure 1.**
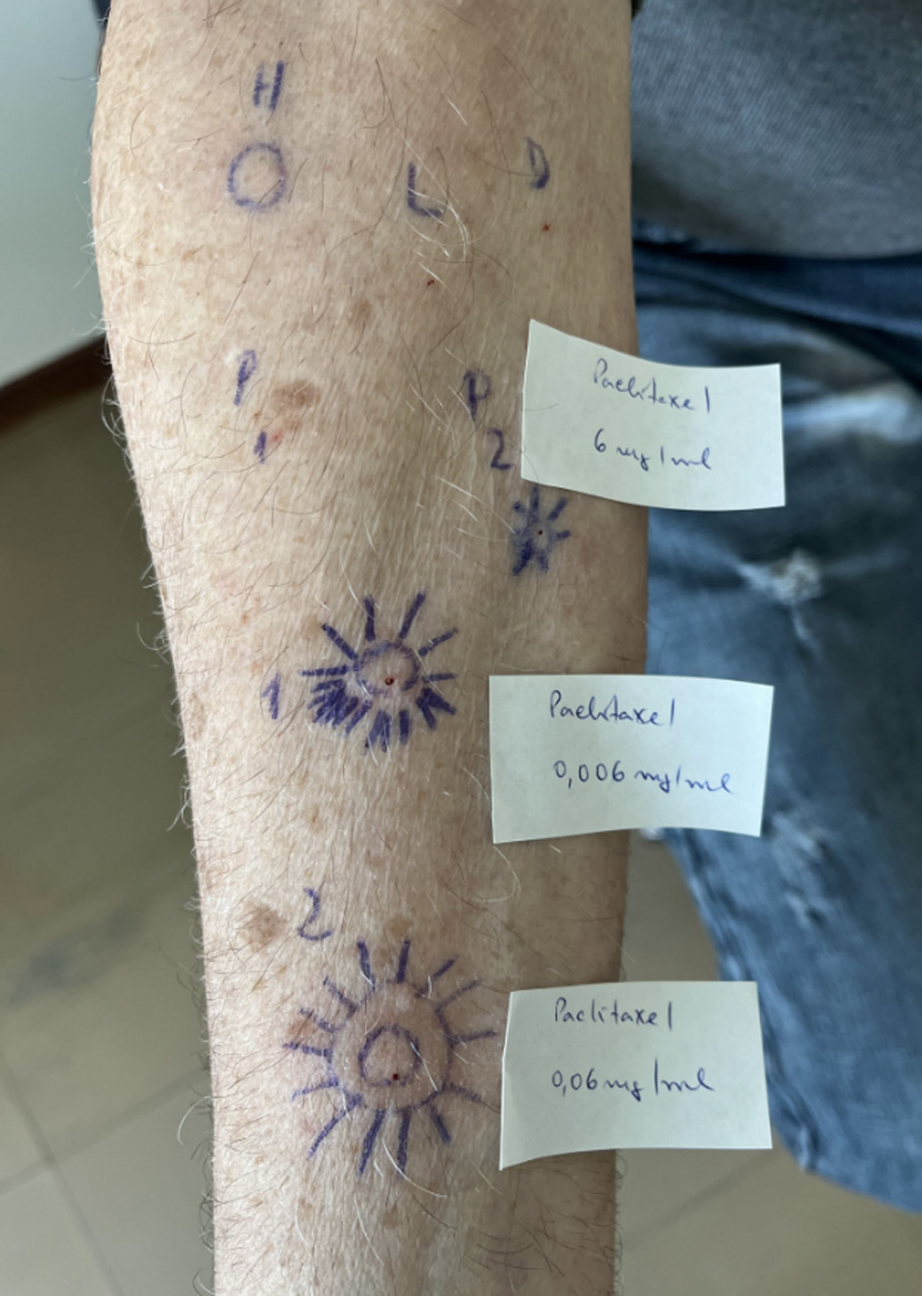
Positive skin sensitivity tests to paclitaxel: prick—0.6 mg/mL and intradermal test at dilutions 1/100 and 1/1000, with 19 mm × 9 mm and 20 mm × 18 mm dimensions, respectively.

Skin prick tests (SPTs) and intradermal tests (IDTs) with paclitaxel were performed. Although SPTs at 0.6 and 6 mg/mL were negative, IDT was positive at 0.06 mg/mL (Fig. 1). While nonirritant concentrations are not unanimously defined in the literature [6], the European Academy of Allergy and Clinical Immunology recommendation of 0.6 mg/mL as the maximum nonirritant concentration for IDTs was followed [7, 8]. In this context, severe anaphylaxis and positive IDT result support an IgE-mediated hypersensitivity. Unlike irritant reactions, HSRs typically elicit responses at lower concentrations, reinforcing the validity of our IDT result.

The positive IDT raised concerns about the patient’s sensitization, considering his French background and previous residence in areas with *Taxus baccata*. Prior exposure to yew pollen may have triggered cross-reactive IgE antibodies, predisposing the patient to an IgE-mediated hypersensitivity to paclitaxel on reexposure.

Desensitization with nab-paclitaxel, a Cremophor EL-free formulation associated with a lower HSR risk [5], was proposed based on the suspected IgE-mediated mechanism, and the oncologist’s decision to adjust the taxane regimen for cancer treatment.

Nab-paclitaxel was administered via a 17-step desensitization protocol with premedication (corticosteroids, antihistamines, montelukast, and acetylsalicylic acid; Table 1) [9, 10]. The protocol was completed without incident, and nab-paclitaxel was well tolerated over 6 cycles without further HSRs. The protocol was not shortened to prioritize safety, allowing uninterrupted lung cancer treatment under close monitoring.

**Table 1.** Sixteen-step paclitaxel desensitization protocol

Step	Dose administered, mg	Concentration, mg/mL	Infusion rate, mL/h	Time, min	Volume, mL
1	0.000504	0.0008*	2.5	15	0.63
2	0.001008	0.0008	5	15	1.25
3	0.002016	0.0008	10	15	2.5
4	0.005	0.0008	25	15	6.25
5	0.01	0.0008	50	15	12.5
6 (TS)	0.02	0.0008/0.008	100	15	25
7	0.05	0.008†	25	15	6.25
8	0.1	0.008	50	15	12.5
9 (TS)	0.2	0.008/0.08	100	15	25
10	0.5	0.08‡	25	15	6.25
11	1	0.08	50	15	12.5
12 (TS)	2	0.08/0.8	100	15	25
13	4	0.8§	20	15	5
14	8	0.8	40	15	10
15	32	0.8	80	30	40
16	40	0.8	100	30	50
17	112	0.8	120	70	140

TS, transition step.

* 0.0008 mg/mL (48.13 mL = 0.038504 mg).

† 0.008 mg/mL (43.75 mL = 0.35 mg).

‡ 0.08 mg/mL (43.75 mL = 3.5 mg).

§ 0.8 mg/mL (245 mL = 196 mg).

## 3. Discussion

This case challenges the traditional understanding of paclitaxel-induced HSRs, suggesting an IgE-mediated mechanism, as indicated by the positive IDT and clinical presentation. Additionally, the patient’s potential environmental sensitization to taxanes through *Taxus baccata* pollen, based on his geographic history, aligns with recent reports of primary sensitization via environmental exposure [11]. This introduces a novel perspective, highlighting the importance of considering environmental factors when assessing taxanes HSRs.

The patient initially experienced a delayed reaction to paclitaxel administration, which was later followed by an anaphylactic shock. This progression aligns with the concept of a “converter phenotype,” as described in recent literature [12]. Patients with this phenotype first exhibit nonimmediate HSRs but subsequently develop immediate reactions upon re-exposure. In this case, prior sensitization to *Taxus baccata* pollen may have predisposed the patient to IgE cross-reactivity with paclitaxel, consistent with the characteristics of the converter phenotype.

However, it is important to consider alternative explanations for the positive skin test response. Non-IgE-mediated mechanisms, such as direct mast cell degranulation via MRGPRX2, that have been implicated in HSRs to opiates and quinolones may also play a role in taxane-induced reactions [13]. The IDT concentration followed guidelines to minimize irritation, but a direct activating effect of the taxane or excipient on mast cells remains possible, potentially causing false positives. While the case suggests an IgE-mediated reaction, confirmatory tests like serum-specific IgE or basophil activation were not conducted, limiting definitive attribution [14]. Future studies with these tests could access their role in taxane-induced HSRs [15].

While nab-paclitaxel is often a safe alternative to paclitaxel-induced reactions and typically used either directly or through a previous drug provocation test [16, 17], desensitization was considered the safer option in this case. Given the patient’s severe reaction and the inability to rule out an IgE-mediated mechanism, desensitization was preferred due to the potential cross-reactivity.

Nevertheless, the role of Cremophor EL in this case cannot be disregarded, but skin testing is challenging to obtain. Given the switch to nab-paclitaxel (which lacks Cremophor EL) and the urgency of treatment, testing seemed less relevant. The priority was to ensure timely therapy, so desensitization was pursued. Future cases may benefit from testing if conventional paclitaxel is used.

Our case underscores the importance of recognizing IgE-mediated hypersensitivity in taxane chemotherapy, particularly in patients with prior yew tree pollen exposure, which is not routinely considered. Skin testing is vital for identifying IgE-mediated HSRs, guiding risk stratification, and determining whether desensitization is necessary. While it remains unclear if the patient could have tolerated nab-paclitaxel without desensitization, the positive IDT results supported a cautious approach.

 In conclusion, this case highlights the need for further research into primary routes of sensitization to taxanes and the optimization of desensitization protocols with a better comprehension of both IgE- and non-IgE-mediated hypersensitivity pathways. Overall, desensitization remains a valuable strategy for enabling safe and effective taxane therapy in patients with taxane HSRs.

## Conflicts of interest

The authors have no financial conflicts of interest.

## Author contributions

JCL: conceptualization, investigation, and writing—original draft. HPP: conceptualization, investigation, and writing—original draft. CMG: writing—original draft. CR: investigation, conceptualization, and writing—review and editing. ATB: supervision and validation. All authors read and approved the final manuscript.
